# Impacts investigation of gas barrier on effective thermal conductivity and service life of vacuum insulation panel

**DOI:** 10.1038/s41598-023-44929-3

**Published:** 2023-11-16

**Authors:** Mingxiao Shi, Lixia Yang, Zhaofeng Chen, Ankang Kan, Shijie Chen, Tianhao He, Jiaxiang Zhang

**Affiliations:** 1 College of Material Science and Technology, Nanjing University of Aeronautics and Astronautics, Nanjing, 211106 People’s Republic of China; 2 Merchant Marine College, Shanghai Maritime University, Shanghai, 201306 People’s Republic of China

**Keywords:** Composites, Materials science, Computational methods

## Abstract

Vacuum Insulation Panels (VIPs) are highly efficient thermal insulation materials with extremely low thermal conductivity based on the vacuum principle. With the sealing properties of the gas barrier envelopes, a long service life of the VIP is obtained. The mechanism and influence factors of gas and water vapor permeability were mathematically analyzed to explore the influence of gas barrier envelopes on the thermal performance of VIPs. Three typical gas barriers were studied, and the selection of the gas barrier and other aspects of optimization were involved. The relationships among temperature, humidity, solubility coefficient, diffusion coefficient, and permeability were concluded, which shows that temperature has a much greater effect on the permeability of the gas barrier relative to humidity. The numerical analysis and influencing factors of VIPs’ service life were also exemplified with three different types of gas barrier envelopes. The experimental results show that depending on the environment, the temperature has a major impact on the effective thermal conductivity and service life of VIP. The research was significant in the selection of gas barriers, the optimization of the performance, and the development of vacuum insulation material.

## Introduction

**Figure 1:**
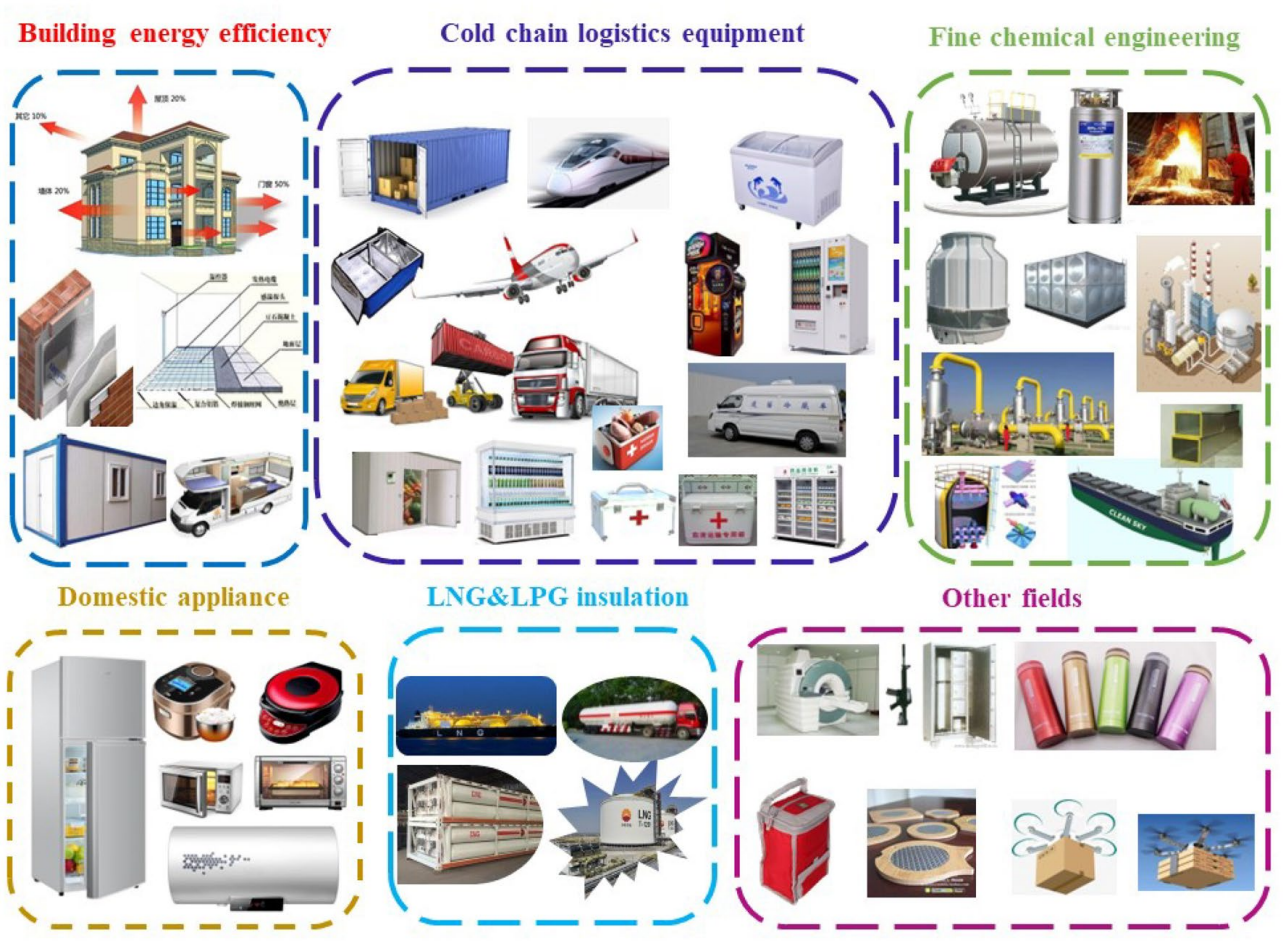
Application fields of VIPs.

In recent years, the issue of global warming has garnered significant attention and has emerged as a topic of considerable interest and concern within the global scientific community and wider society. The likelihood of experiencing extreme weather events has been steadily increasing, with summers becoming hotter and winters growing colder. In response, there has been a corresponding surge in energy consumption, particularly for cooling and heating systems, to maintain acomfortable living environment. This has resulted in a significant rise in carbon emissions, thereby exacerbating the greenhouse effect and posing formidable challenges for the future development of human society. Ever since China's successful implementation of its carbon peak and carbon neutrality strategy, the nation has witnessed a remarkable surge in the popularity and growth of its low-carbon economy. The development of green energy and energy-saving materials has been accorded the utmost priority, as the country continues its steadfast commitment towards achieving a more sustainable and eco-friendly future^1^. Figure 1 illustrates the extensive applications of Vacuum Insulated Panels (VIPs) in areas such as building energy conservation, cold chain logistics, fine chemicals, household appliances, etc. VIPs have emerged as a highly efficient and promising energy-saving material, with vast prospects for further development.

**Figure 2:**
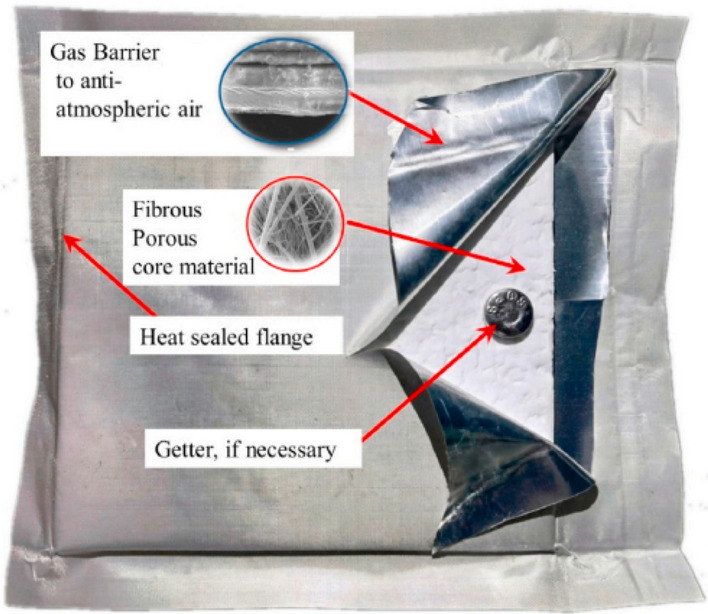
Schematic of VIP^6^.

Figure 2 shows the schematic of VIP, which is composed of three parts: gas barrier, porous core material, and getter. The core material is input into the gas barrier envelopes and then vacuum sealed to obtain a high vacuum internal structure, the thermal conductivity of VIP can be as low as 2mW/(m**·**K), which is the lowest of thermal insulation materials^2–5^. With energy conservation and environmental protection advantages, VIP is increasingly widely used.

The thermal conductivity of the core material determines the thermal insulation performance of the VIP to a certain extent. Porous materials such as fumed silica, aerogel, glass fiber, and microporous polyurethane are generally selected as core materials. Taking the ultra-fine glass fiber core material as an example, on the one hand, the fiber staggered lap leads to a significant increase in the heat conduction path, so the heat conduction coefficient is effectively reduced. On the other hand, the pore size formed by the micron super-fine glass fiber lap is small, and the size of a nanometer can be formed after vacuum suction, which can effectively eliminate the convective heat transfer, to obtain a low thermal conductivity^7, 8^.

**Figure 3:**
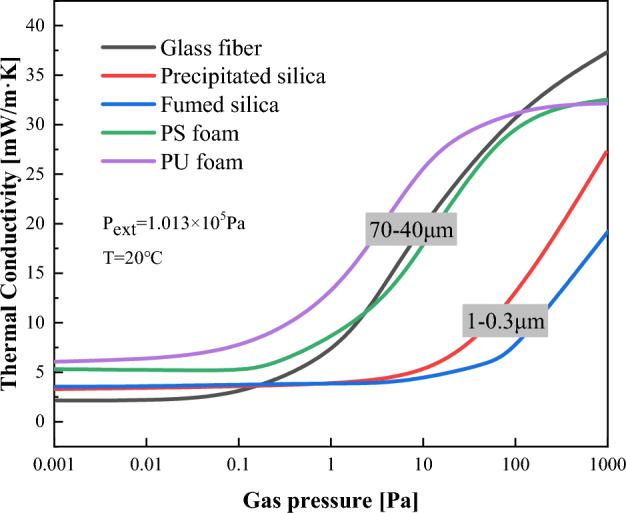
Relationship between the inner pressure and thermal conductivity of VIPs (redrawn from^9^).

The unique vacuum structure inside the VIP is the key to its low thermal conductivity. To ensure the thermal insulation performance of the VIP over a long time, it is very important to maintain the stability of the vacuum degree in the VIP. As shown in Fig. 3, the sensitivity of different core materials to pressure is different, resulting in great differences in their thermal insulation performance under different vacuum degrees^10–12^.

As the packaging material of the VIP, the gas barrier has a certain degree of gas resistance and water vapor resistance but is not airtight. Gas can penetrate the VIP through the surface of the gas barrier, or dissolve into the gas barrier through adsorption-diffusion, which will greatly affect the vacuum degree of the VIP, and then affect the long-term stability of its thermal insulation performance^13^. Zhou^14^ designed a vacuum insulation array (VIA) with independent vacuum units, which can achieve local damage without affecting the thermal insulation properties of the overall structure, so it can be pierced and cut or reassembled, which is a breakthrough in the field of VIP research, but still cannot avoid fatigue damage of gas barriers under long-term use conditions. Therefore, to improve the thermal performance of VIPs, it is necessary to study and optimize the performance of the gas barrier.

There is much theoretical research on gas barriers. Hubert Schwab and Ulrich Heinemann^15^ studied the permeability of oxygen and water vapor of AF, MF1, and MF2 gas barriers under different VIP specifications and humidity. The results showed that if the boundary of the VIP is increased by 2 times, the pressure growth rate in the VIP would be reduced by half. Water vapor permeability is closely related to the core material’s water absorption characteristics and the environment’s relative humidity. S. Brunner^16^ adopted Fib-Etching technology to study the influence of different aluminum thicknesses on reducing gas and water vapor permeability. Wang^17^ discussed a new type of low thermal conductivity and high-temperature vacuum insulation composite composed of a C_*f*_/SiC sealing shell, which has great application potential as a thermal protection system material. Hubert and Similler^18, 19^ also did a lot of related research on the influence of gas penetration of barrier membranes on the service life of VIPs and put forward the gas permeability performance index of VIPs in different areas within the required service life range. Zhang^20^ studied in detail the performance of gas barriers and the influence of boundary thermal effect on the performance of VIPs and successfully developed a nano gas barrier that replaced aluminum foil. Li^21^ systematically analyzed the factors affecting the performance of gas barriers, discussed the influence of the thermal bridge effect, gas penetration, and the size on the performance of VIPs discussed the ways and requirements to reduce the thermal bridge effect and gas penetration, and summarized the methods to improve the performance of the gas barrier. In their research, Oliver Miesbauer^22^ proposed a new idea of opaque high-barrier films. The barrier and mechanical properties of these films and their applicability in construction are discussed. Li^23^ used three films (AF, MF, and GF) and VIPs (thermal insulation core material-precipitated silica) using these three films for the degradation study. The effects of temperature, alkali, and local stress concentration on the properties of gas barriers were discussed. Glenn De Meersman^24^ studied the experimental process of VIP degradation under different humid and hot conditions at room temperature and controlled climate chambers and determined the Sd value of aluminum cladding based on these measurements. Martin^25^. explained the reasons for the different thermal resistance values of reflective multi-foil insulations under different test methods and introduced the standard calculation procedure for calculating the thermal resistance of reflective multi-foil insulations. The studies mentioned above are mostly the adjustment of different macro parameters and their impact on the service life of VIPs, such as VIP size, gas barrier thickness, gas barrier type, etc., but the research on the mechanism of the gas barrier permeation is minimal. To ensure the long-term stability of VIPs’ thermal insulation performance, improving the performance of the gas barrier in principle is an important issue that needs to be studied urgently.

In this paper, the principle and influencing factors of the permeability of the gas barrier are analyzed, and the mathematical model is established to study the influence results in theory, which is of great significance to optimizing the gas barrier’s performance and extending the service life of VIPs.

## The permeability of the gas barrier

### Penetration mechanism

**Figure 4:**
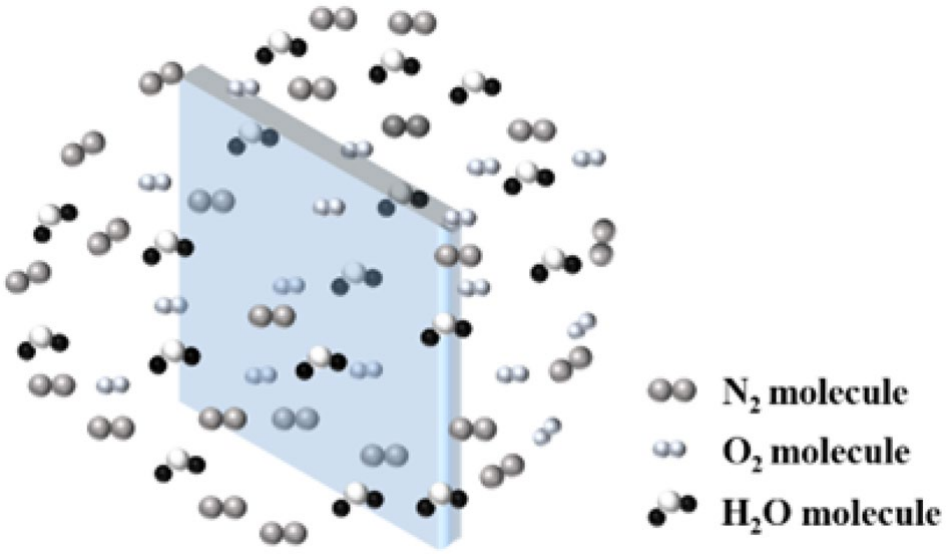
Schematic diagram of the gas barrier permeation process.

The gas barrier is composed of polymer materials with gas and water vapor resistance but is not airtight. As shown in Fig. 4, gaseous molecules such as oxygen, nitrogen, and water vapor present in the service environment can permeate through the gas barrier and infiltrate the interior of VIPs, leading to a disruption in the vacuum integrity of VIPs and consequently exerting a detrimental impact on thermal insulation performance. Most scholars believe that the penetration process of gas to the gas barrier is a single-molecule diffusion process, which belongs to the mass transfer process^26^. When the gas penetrates the gas barrier, it goes through three continuous processes: (1) the dissolution of the gas on the surface of the gas barrier, (2) the diffusion of the gas from high concentration to low concentration in the gas barrier, (3) the solution of the gas from the surface on the other side of the gas barrier. There is no chemical reaction between the gas barrier and the gas, and the internal and external environment of the gas barrier will be in dynamic equilibrium when the permeation process is completed^27^.

The permeability coefficient, a measure of a gas barrier's ability to resist gas penetration, is determined by the dissolution-diffusion mechanism. According to this mechanism, the gas permeability coefficient can be expressed as the product of the gas solubility coefficient, which reflects the ability of a gas to dissolve in the barrier material, and the gas diffusion coefficient, which reflects the ability of a gas to diffuse through the barrier material^28^:

1 $$P=S\times D$$

where *P* is the permeability coefficient, *S* is the solubility coefficient, and *D* is the diffusion coefficient. It can be seen from Eq. (1) that the permeability coefficient of the gas is related to the solubility coefficient *S* and diffusion coefficient *D*, so the permeability of the gas to the gas barrier depends on its diffusion ability and solubility ability.

In a broad sense, the permeability coefficient of the gas barrier can be expressed by Eq. (2):

2 $$P={F}_{i}\times {G}_{i}\times {A}_{k}$$

where ${F}_{i}$ is the influence coefficient of the gas barrier properties on permeability, ${G}_{i}$ is the influence coefficient of gas characteristics on permeability, and ${A}_{k}$ is the influence of environmental and other factors on permeability. It can be seen from Eq. (2) that the permeability coefficient of the gas barrier is affected by many factors, ${F}_{i}$ and ${G}_{i}$ are the essential parameters of the gas barrier and the permeable gas, and their values depend on the material of the gas barrier and the type of gas, which is not discussed here, the influence of environmental factors on the permeability of the gas barrier ${A}_{k}$ is only considered in this work. Based on the permeability mechanism, the relationship between temperature and humidity and the solubility coefficient S and diffusion coefficient D of the gas barrier is studied by numerical analysis, so that the influence of temperature and humidity on the permeability of the gas barrier can be effectively obtained.

### Effect of temperature on the permeability

The solubility *S* is a parameter indicating the solubility of the gas, which is related to the gas properties, the gas barrier structure, the ambient temperature and humidity, and the gas pressure. The diffusion coefficient *D* is a parameter indicating the diffusion capacity of the gas, which is related to the type of gas and the ambient temperature and pressure. According to Arrhenius law^29^, the solubility coefficient S and diffusion coefficient *D* of the barrier membrane as a function of temperature can be expressed by Eqs. (3) and (4):

3 $$S={S}_{0}exp(-\frac{{E}_{s}}{RT})$$

4 $$D={D}_{0}exp(-\frac{{E}_{D}}{RT})$$

where ${S}_{0}$ is the limit of the* S* of the gas barrier when the temperature goes to 0, ${D}_{0}$ is the limit of the* D* of the gas barrier when the temperature goes to 0, ${E}_{s}$ and ${E}_{D}$ are solution activation energy and diffusion activation energy respectively, *R* is the gas constant, and *T* is the absolute temperature.

The law is also followed for the permeability coefficient *P*:

5 $$P={P}_{0}exp(-\frac{{E}_{P}}{RT})$$

where ${P}_{0}$ is the limit of the *P* of the gas barrier when *T* goes to 0, ${E}_{P}$ is the permeation activation energy.

According to Eq. (1):

6 $${P}_{0}={S}_{0}\times {D}_{0}$$

7 $${E}_{P}={E}_{S}+{E}_{D}$$

8 $$P={S}_{0}{D}_{0}exp(-\frac{{E}_{S}+{E}_{D}}{RT})$$

In general, the diffusion coefficient *D* increases with the increase in temperature, and the solution coefficient *S* of the gas varies with the characteristics of the permeate gas. The ${E}_{s}$ is positive for invariant gases (H_2_, N_2_, O_2_, etc.), and the *S* increases with increasing temperature. For condensable gas (water vapor), the ${E}_{S}$ is negative, and the *S* decreases with the increase in temperature.

Table 1 lists the water vapor permeability of PET12μm, PET20μm, and aluminized PET18μm at different temperatures tested by the Gas Transmission Rate Tester, which verifies the correctness of the theoretical analysis. From another point of view, the diffusion of gas molecules is actually with the help of the free volume or hole in the gas barrier density fluctuation appears as a channel, the gas barrier is generally composed of polymer, with the increase of temperature, the more the polymer expands, the more the polymer chain moves, resulting in the more opportunities for the appearance of such holes, and therefore the greater the permeability of the gas barrier.

**Table 1:** Water vapor permeability of three gas barrier materials at various temperatures.

Temperature (°C)	Humidity (%RH)	Water vapor permeability (g/cm^2^·24 h)		
		PET12μm	PET20μm	Aluminized PET18μm
22	90	8.79	3.95	1.32
24		10.75	4.63	1.46
26		11.79	6.40	1.79
28		12.83	6.83	1.88
30		14.00	7.17	1.93
32		16.41	8.68	2.21

(Derived from^22^).

### Effect of humidity on the permeability

When the humidity of the environment rises, the moisture in the environment will diffuse into the gas barrier, and the penetration of this moisture is equivalent to the addition of additives to the material, which plasticizes the polymer^30, 31^. This plasticizing effect increases the number of holes in the film and makes the diffusion of the gas easier and faster. The effects of humidity and plasticization on gas barrier permeability can be expressed by Eq. (9):

9 $${e}^{\eta \times {X}_{w}}={e}^{\eta \times S\times {P}_{SAT}\times \overline{RH} }$$

where $\eta$ is the plasticity coefficient, ${X}_{w}$ is the water vapor content, ${P}_{SAT}$ is the saturation pressure, and $\overline{RH }$ is the average relative humidity on both sides of the gas barrier. The effect of humidity on the permeability coefficient is different for different materials, when the humidity increases, the permeability coefficient of most materials will increase, and which of individual materials will decrease.

There are also some gas barriers whose permeability coefficients change slightly, as shown in Table 2, mainly because the composition of these gas barriers is strong active molecules, the transmittance of the gas is almost not affected by the humidity, for the VIP, PE, and PET as the main component of its gas barrier is typical strong active molecules, so humidity has little effect on the permeability coefficient, which is far less than the influence of temperature. Therefore, the influence of temperature on the permeability of the gas barrier is mainly considered in the next work.

**Table 2:** Water vapor permeability of three gas barrier materials at various humidity.

Material	Temperature (°C)	Humidity (%RH)	Water vapor permeability (g/cm^2^·24 h)
PET12μm	20.1	80.4	8.93
	20.1	85.1	9.05
	20.0	88.8	9.05
	20.2	90.1	9.07
PET20μm	32.3	88.6	6.79
	32.6	88.9	8.68
	32.6	91.3	7.36
	32.8	92.1	7.54
Aluminized PET18μm	28.2	88.3	1.88
	28.3	89.9	1.41
	28.6	90.0	2.35
	28.9	91.3	1.54

(Derived from^22^).

## The service life of VIP

### Theoretical model

**Figure 5:**
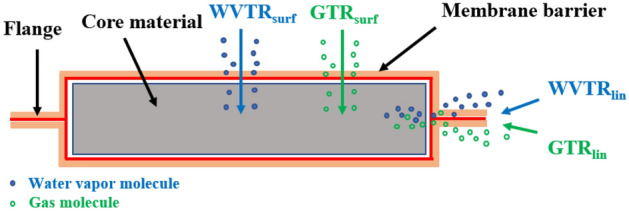
Permeation model of VIP.

Figure 5 shows the mechanism of the permeation of water vapor and gas during the application of the VIP. The permeability of gas and water vapor through the gas barrier will lead to the increase of the VIP thermal conductivity, when the thermal conductivity exceeds a critical value, the VIP will be considered as failed. The American standard ASTM C 1484-01 stipulates that this value is 11.5 mW/(m·K), and the service life of the VIP is the corresponding use time in the event of failure.

The thermal conductivity of the VIP is mainly affected by temperature, humidity, and pressure^32^, to predict the service life of the VIP, it is generally considered that the influence of gas permeation and water vapor permeation on the thermal conductivity of the VIP is independent, which can be obtained by Eq. (10)^18^

10 $${\lambda }_{ETC}={\lambda }_{evac}+{\lambda }_{gas}\left({P}_{gas}\right)+{\lambda }_{w}\left({X}_{w}\right)$$

where ${\lambda }_{\mathrm{ETC}}$ is the effective thermal conductivity of the VIP after use, ${\lambda }_{evac}$ is the initial thermal conductivity of the VIP, ${\lambda }_{gas}\left({P}_{gas}\right)$ is the added value of the VIP thermal conductivity due to gas permeation, and ${\lambda }_{w}\left({X}_{w}\right)$ is the added value of the VIP thermal conductivity due to water vapor permeation.

${\lambda }_{gas}\left({P}_{gas}\right)$ can be expressed by Eq. (11)^33^:

11 $${\lambda }_{gas}\left({P}_{gas}\right)=\frac{{\lambda }_{g0}}{(1+{P}_{1/2}/{P}_{gas})}$$

where ${\lambda }_{g0}$ is the thermal conductivity of free static air, which is 26mW/(m·K), ${P}_{1/2}$ is the corresponding pressure when the thermal conductivity of the gas becomes ${\lambda }_{g0}/2$, which mainly depends on the pore size of the core material and the type of the gas, ${P}_{gas}$ is the internal pressure of the VIP.

${\lambda }_{w}\left({X}_{w}\right)$ can be expressed by Eq. (12)^34^:

12 $${\lambda }_{w}\left({X}_{w}\right)=b\times {X}_{w}$$

where *b* is the tested coefficient related to the properties of the core material, ${X}_{w}$ is the humidity of the VIP.

Equation (13) and Eq. (14)^35^ conclude the relationship between ${P}_{gas}$ and ${X}_{w}$ over time:

13 $${P}_{gas}(t)=\frac{{\text{GTR}}_{\text{tot}}}{{V}_{\text{eff}}}(\frac{{T}_{m}{P}_{0}}{{T}_{0}})t$$

14 $${X}_{w}\left(t\right)=f\times {k}_{out}\times \left(1-\mathit{exp}\left(\frac{{WVTR}_{tot}}{{m}_{dry}\times f}\times t\right)\right)$$

where ${\text{GTR}}_{\text{tot}}$ is the total gas permeability, ${V}_{\text{eff}}$ is the effective pore volume of the VIP core material, ${T}_{m}$ is the test temperature, ${T}_{0}$ is the standard temperature, ${P}_{0}$ is the standard atmospheric pressure, $f$ is a constant related to the characteristics of the core material, ${k}_{out}$ is the humidity of the environment, ${WVTR}_{tot}$ is the total water vapor permeability, t is the time.

Equation (10) can be further deduced as:

15 $${\lambda }_{ETC}={\lambda }_{evac}+\frac{{\lambda }_{g0}}{(1+{P}_{1/2}/(\frac{{\text{GTR}}_{\text{tot}}}{{V}_{\text{eff}}}\left(\frac{{T}_{m}{P}_{0}}{{T}_{0}}\right)t)}+b\times f\times {k}_{out}\times \left(1-\mathit{exp}\left(\frac{{WVTR}_{tot}}{{m}_{dry}\times f}\times t\right)\right)$$

The service life of the VIP can be predicted by Eq. (15). It also shows that in addition to core material characteristics and other factors, the permeability of the gas barrier also affects the service life of the VIP. Therefore, the influence of temperature on the service life of the VIP will be studied in the following work to verify the accuracy of the numerical analysis of the permeability of the gas barrier, which will have important guiding significance for the choice of the gas barrier and the application of the VIP.

### Experimental methods

**Figure 6:**
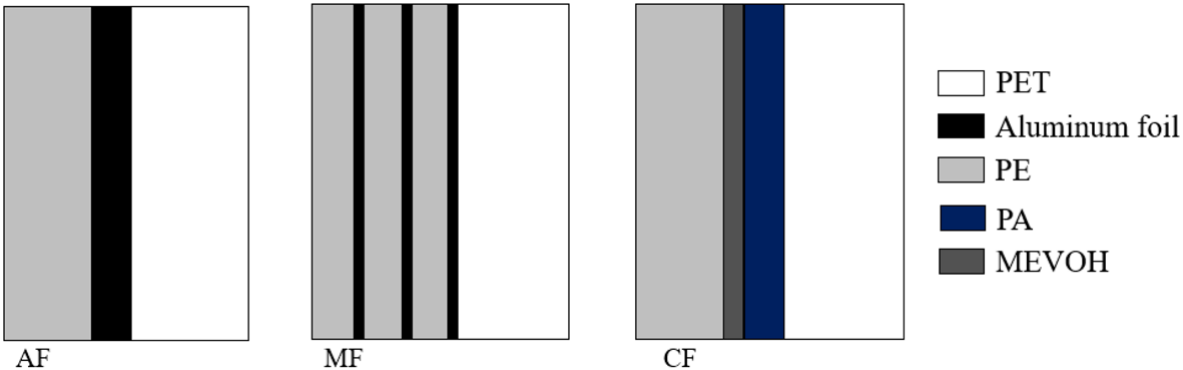
Composition of AF(Al-foil), MF(Al-Metallized film), and CF(Composite film) gas barriers.

Three kinds of VIPs with different gas barrier envelopes were prepared in this work, with glass fiber as the core material, the size of each VIP is 300 mm × 300 mm × 10 mm. The structure of the three gas barriers is shown in Fig. 6, and the permeation parameters are shown in Table 3.

**Table 3:** The permeation parameters of the gas barriers.

Gas barrier type	Oxygen permeability cm^3^/(m^2^ 24 h)	Water vapor permeability g/(m^2^ 24 h)
AF	0.0051	0.0091
MF	0.0072	0.0103
CF	0.0093	0.0121

**Figure 7:**
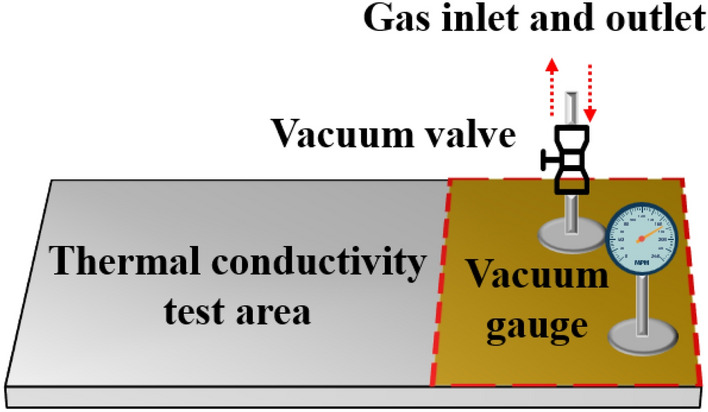
Diagrammatic representation of the experimental setup for exploring the correlation between the internal pressure of vacuum insulation panels (VIPs) and their effective thermal conductivity.

Figure 7 is the schematic diagram of the test device for the relationship between VIP internal pressure and effective thermal conductivity. First, a vacuum pump is used to connect the gas inlet and outlet, after holding the pressure at 0.1 Pa for 10 min, the vacuum valve is closed immediately, and the initial thermal conductivity of VIPs can be measured when the vacuum gauge is stabilized. Afterward, by controlling the opening and closing of the vacuum valve, the effective thermal conductivity of VIPs under different internal pressures can be measured, providing a reference for predicting the service life of VIPs.

**Figure 8:**
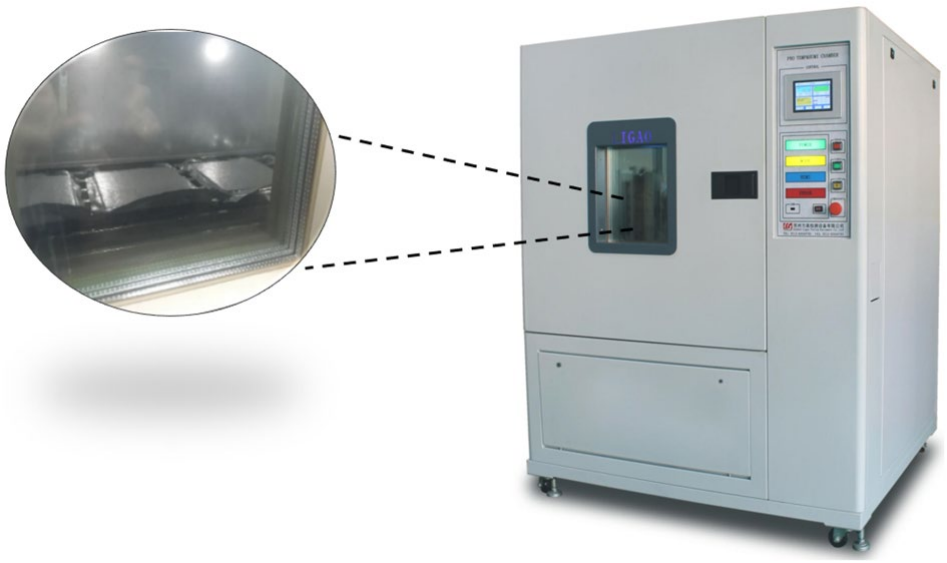
High-low pressure/high-low temperature test chamber.

As shown in Fig. 8, VIP samples named AF-VIP, MF-VIP, and CF-VIP, were placed in high-low pressure/high-low temperature test chamber (20 °C, 70 °C, 120 °C, and 140 °C, 90% RH), taken out, and measured the effective thermal conductivity of VIPs every 7 days, with a period of 4 weeks. The effect of temperature on VIP performance can be derived by observing the change in the effective thermal conductivity of VIPs.

### Experimental results

**Figure 9:**
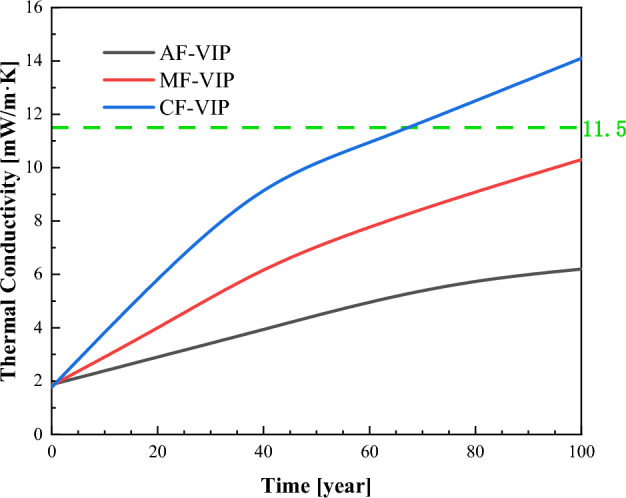
Theoretical prediction for the service life of the three VIPs.

According to the VIP service life theory model, combined with the VIP internal pressure and effective thermal conductivity test device, the service life of the three VIPs is predicted, VIPs’ service environment is assumed at standard atmospheric pressure, 20 °C, and 50% RH. As shown in Fig. 9, the green line is the thermal conductivity when the VIP fails. It can be observed that AF-VIP has the longest service life of more than 100 years, MF-VIP is second, and CF-VIP is the shortest, only about 70 years.

The difference between them has a crucial relationship with the composition of the gas barrier. Figure 6 has given the composition structure of three types of gas barriers, of which the aluminum foil layer in the AF gas barrier is the thickest, and has the best barrier performance, but the over-thick aluminum foil layer will lead to the thermal bridge effect, which is also the reason why the initial thermal conductivity of AF-VIP is higher than that of the other two VIPs. The MF gas barrier with a multi-layer aluminum coated film can significantly reduce the effect of the thermal bridge effect but leads to a decrease in barrier performance. As a non-metalized film, CF barrier film has the lowest thermal bridge effect, and CF-VIP has the lowest thermal conductivity, but its service life is poor. This is because the core barrier layer in the CF gas barrier is the MEVOH layer. MEVOH has a high barrier to non-polar molecules such as oxygen, but is particularly sensitive to water molecules and thus exhibits high permeability, increasing VIPs’ thermal conductivity and decreasing service life.

**Figure 10:**
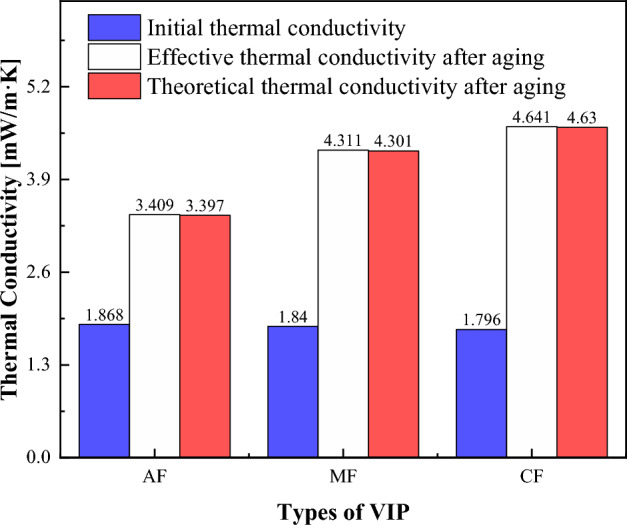
Comparison of effective and theoretical thermal conductivity at 70 °C and 90% RH.

To compare the accuracy of theoretical models, the effective thermal conductivity of three VIPs after aging at a temperature of 70 °C and 90% RH was obtained through experimental tests and theoretical predictions. Figure 10 illustrates a minor deviation between the theoretically calculated thermal conductivity and the actual effective thermal conductivity. However, this deviation falls well within the acceptable range, affirming the reliability and accuracy of the experimental results. The observed discrepancy between the theoretical model and experimental results may be attributed to the volatilization of water within the core material, which had not been accounted for in the theoretical calculations. The core material used in this work is glass fiber, which is prepared by a wet process and will inevitably have moisture residue. The rise in temperature leads to the volatilization of moisture, which emerges as the primary factor contributing to the observed deviation between the theoretical and experimental values.

**Figure 11:**
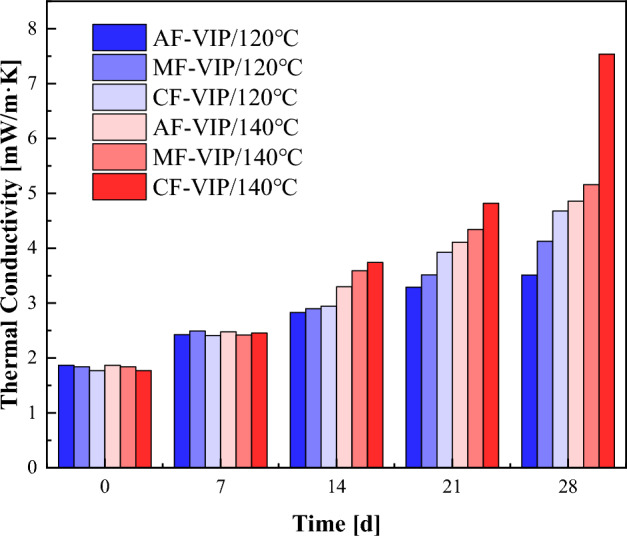
The effective thermal conductivity of three VIPs at 120 °C and 140 °C, 90% RH.

It can be seen from Fig. 10 that the effective thermal conductivity of the three VIPs shows an increasing trend with the increase in temperature, and with further increase in temperature, the effective thermal conductivity of VIPs varies more significantly over time, as shown in Fig. 11, especially for CF-VIP, which fits well with the theoretical model. The permeability of the gas barrier is the main reason for the change in the thermal conductivity of VIPs, so the effective thermal conductivity of CF-VIP changes the most. The high temperature will exacerbate this trend of change, because the pinhole defect in the gas barrier will expand outward under the influence of high temperature, forming a diffusion channel, accelerating the rapid diffusion of gas and water vapor, resulting in increased permeability of the gas barrier and a decrease in air tightness^36^, thereby increasing the effective thermal conductivity of VIPs, which will accelerate the failure rate of VIPs, thus affecting the service life of VIPs. Therefore, it’s necessary to choose gas barriers with better performance to cope with complex environments in practical applications, which has important guiding significance for the application of VIPs.

## Conclusions

The relevant factors affecting VIP were systematically studied through numerical analysis and experimental methods, combined with molecular motion dynamics and the Arrhenius Law, leading to the following conclusions being reached:

1. Temperature has a greater impact on the permeability of the gas barrier, while humidity has a lesser impact on the permeability of the gas barrier.
2. With the increase in temperature, the effective thermal conductivity of the VIP shows a continuously rising trend. In addition, prolonged exposure to high temperatures accelerates VIP failure, which significantly reduces its service life.
3. The permeability of the gas barrier directly affects the stability of the thermal insulation performance of the VIP, and the selection of a good gas barrier helps to deal with the influence mechanism of the complex environment on the permeability and extend the service life of the VIP.

## Data Availability

The datasets used and/or analyzed during the current study are available from the corresponding author on reasonable request.

## List of symbols

P: Permeability coefficient
S: Solubility coefficient
D: Diffusion coefficient
${F}_{i}$: Influence coefficient of the gas barrier properties on permeability
${G}_{i}$: Influence coefficient of gas characteristics on permeability
${A}_{k}$: Influence of environmental and other factors on permeability
${S}_{0}$: Limit of the* S* of the gas barrier when the temperature goes to 0
${D}_{0}$: Limit of the* D* of the gas barrier when the temperature goes to 0
${E}_{s}$: Solution activation energy
${E}_{D}$: Diffusion activation energy, kJ/mol
R: Gas constant
T: Absolute temperature, K
${P}_{0}$: Limit of the *P* of the gas barrier when *T* goes to 0
${E}_{P}$: The permeation activation energy, kJ/mol
$${X}_{w}$$: Humidity
${P}_{SAT}$: Saturation pressure, Pa
$\overline{RH }$: Average relative humidity
${P}_{gas}$: Internal pressure, Pa
b: Tested coefficient related to the properties of the core material
${\text{GTR}}_{\text{tot}}$: Total gas permeability, g/cm^2^·24 h
*V*_*eff*_: Effective pore volume, m^3^
$${T}_{m}$$: Test temperature, K
$${T}_{0}$$: Standard temperature, K
${P}_{0}$: Standard atmospheric pressure, Pa
$f$: Constant related to the characteristics of the core material
${k}_{out}$: Humidity of the environment
${WVTR}_{tot}$: Total water vapor permeability, g/cm^2^·24 h
t: Time

## Greek symbols

$$\eta$$: Plasticity coefficient
${\lambda }_{\mathrm{ETC}}$: Effective thermal conductivity, W/(m·K)
${\lambda }_{evac}$: Initial thermal conductivity, VIP, W/(m·K)
${\lambda }_{gas}\left({P}_{gas}\right)$: The added value of thermal conductivity due to gas permeation, W/(m·K)
Pa: ${\lambda }_{w}\left({X}_{w}\right)$ The added value of thermal conductivity due to water vapor permeation, W/(m·K)
${\lambda }_{g0}$: Thermal conductivity of free static air, W/(m·K)

## Abbreviations

VIP: Vacuum Insulation Panel
AF: Al-foil
MF: Al-Metallized film
GF: Glass fiber film
CF: Composite film
